# Measurement Properties and Quality of Maternal Breastfeeding Instruments: A Scoping Review

**DOI:** 10.1177/08903344261430247

**Published:** 2026-04-26

**Authors:** Seyedeh Samira Mokhlesi, Vidanka Vasilevski, Kolsoom Safari, Linda Sweet

**Affiliations:** 1 School of Nursing and Midwifery, Centre for Quality and Patient Safety Research in the Institute for Health Transformation, Deakin University, Burwood, VIC, Australia; 2 Western Health, St Albans, VIC, Australia; 3 School of Nursing and Midwifery, La Trobe University, Bundoora, Melbourne, VIC, Australia

**Keywords:** Breastfeeding, lactation, psychometrics, surveys and questionnaires

## Abstract

**Background:**

Different questionnaires are available to assess breastfeeding behaviour and factors that impact breastfeeding success. However, it is necessary to determine whether the questionnaire’s measurement properties are satisfactory before using them.

**Aim:**

Evaluate, compare, and summarise the measurement properties and quality of the original version of maternal breastfeeding questionnaires.

**Methods:**

This review used the preferred reporting items for systematic reviews and meta-analysis extension for scoping reviews (PRISMA-Scr). Peer-reviewed primary research studies that focused on the development and evaluation of the psychometric properties of breastfeeding instruments were included. The consensus-based standards for the selection of health measurement instrument (COSMIN) methodology was used to assess the methodological quality and measurement properties of the breastfeeding instruments.

**Results:**

From 3,850 abstracts, 25 papers were identified. Only six instruments demonstrated sufficient content validity and internal consistency and were recommended for broad use. The results obtained with these instruments can be trusted. Sixteen of the 25 instruments had evidence of indeterminate (±) content validity. Although they had the potential for recommendation, further research is needed to assess their quality. None of the instruments reported cross-cultural validity/measurement invariance, measurement error, or responsiveness. One instrument was not recommended for use due to insufficient content validity.

**Conclusion:**

The review highlights the need for further research into the measurement properties of most existing breastfeeding instruments, especially cross-cultural validity/measurement invariance, measurement error, and criterion validity.

## Introduction

The World Health Organization (WHO) and the United Nations Children’s Fund recommend exclusively breastfeeding infants for the first 6 months of life, followed by continued breastfeeding for at least 2 years (Adsit & Hewlings, 2022). Breastfeeding has several advantages for the mother and child. Infant health benefits include a lower risk of respiratory infections, asthma, leukaemia, and sudden infant death syndrome (Falcone et al., 2018). Moreover, breastfeeding strengthens the emotional connection between mother and child and promotes infants’ psychological and intellectual development (Moubareck, 2021). Maternal health benefits include a decreased risk of obesity, diabetes, hypertension, myocardial infarction, hyperlipidemia, and breast and ovarian cancer (W. Wu et al., 2021). Despite growing awareness of the benefits of breastfeeding and the associated risks of formula-feeding, breastfeeding rates continue to stagnate. Previous studies indicate that only 39% of infants globally are exclusively breastfed for the first 4 months, and an even smaller percentage are breastfed for the recommended 6 months (Salem & Ertz, 2023).

Breastfeeding success can be influenced by various factors, including the self-efficacy of the mother, breastfeeding attitudes and knowledge, social support, health service support, and factors related to the child’s health (Čatipović et al., 2023; Piankusol et al., 2021). It is a public health priority to assess these factors to promote breastfeeding (Borona et al., 2023). The use of valid and reliable breastfeeding instruments is valuable for improving care and conducting research (Casal et al., 2017). Precise instruments enable healthcare providers to measure factors that impact breastfeeding practices accurately, which can help design personalised education and support strategies tailored to individual needs (Borona et al., 2023). Moreover, these instruments provide evidence to develop effective interventions in research (Xia et al., 2023).

Although numerous questionnaires have been developed to assess breastfeeding behaviour and factors that impact breastfeeding success (Čatipović et al., 2023), the psychometric properties of these instruments vary widely. When using instruments, it is crucial to examine whether the measurement properties are satisfied (Lee et al., 2020). Polit (2015) criticised nursing researchers for having primarily concentrated on classical measurement properties established decades ago by psychometricians, even though new measurement ideas have emerged and been implemented in other healthcare domains. The consensus-based standards for the selection of health measurement instruments (COSMIN) is a standardized tool that assesses the methodological quality and measurement properties of patient-reported outcome measures (PROMs) and offers recommendations for selecting the most suitable instrument (Prinsen et al., 2018).

Although COSMIN has been used to assess the psychometric properties of specific breastfeeding instruments that measure self-efficacy (Borona et al., 2023), a comprehensive evaluation covering all breastfeeding instruments is still needed. Therefore, evaluating the psychometric properties of existing breastfeeding instruments is a gap in the literature. Such a review could facilitate the selection of the most suitable instrument and the development of higher-quality instruments (Xia et al., 2023). For this reason, this study aimed to critically evaluate, compare, and summarise the measurement properties and quality of the original version of breastfeeding questionnaires.

**Key Messages**

• The existing breastfeeding questionnaires need to be carefully evaluated for their methodological quality and measurement properties to choose the most suitable instrument.

• Only six out of 25 reviewed instruments demonstrated sufficient content validity and internal consistency, while most showed indeterminate content validity, highlighting the need for further validation research.

• None of the reviewed breastfeeding instruments assessed cross-cultural validity or measurement invariance, which is essential to ensure accurate and meaningful use across culturally diverse populations.

• This study offers a comprehensive evaluation of breastfeeding questionnaires, helping to select the most reliable tools and identify areas for further research.

## Methods

### Research Design

This scoping review was conducted and reported using the preferred reporting items for systematic reviews and meta-analysis extension for scoping reviews (PRISMA-Scr) (Tricco et al., 2018). The COSMIN methodology was used to assess the measurement properties and quality of the breastfeeding instruments (Prinsen et al., 2018).

### Literature Search

#### Sample: Defining the Articles Reviewed

Peer-reviewed articles dedicated to developing and evaluating the psychometric properties of breastfeeding instruments were included. Inclusion criteria consisted of 1) full-text articles focused on the initial development and validation of breastfeeding assessment instruments; 2) studies that targeted adult parents, pregnant mothers, or breastfeeding mothers with term and healthy babies; and 3) English-language publications (the original tool may have been in another language but translated to English for publication).

Publications were excluded if they focused on the translation and adaptation of existing breastfeeding instruments or studies targeted at healthcare providers, teenage mothers, or mothers of preterm babies. We did not consider editorials, book chapters, guidelines, letters to the editor, nonhuman studies, review articles, or publications in languages other than English.

**Figure 1.**
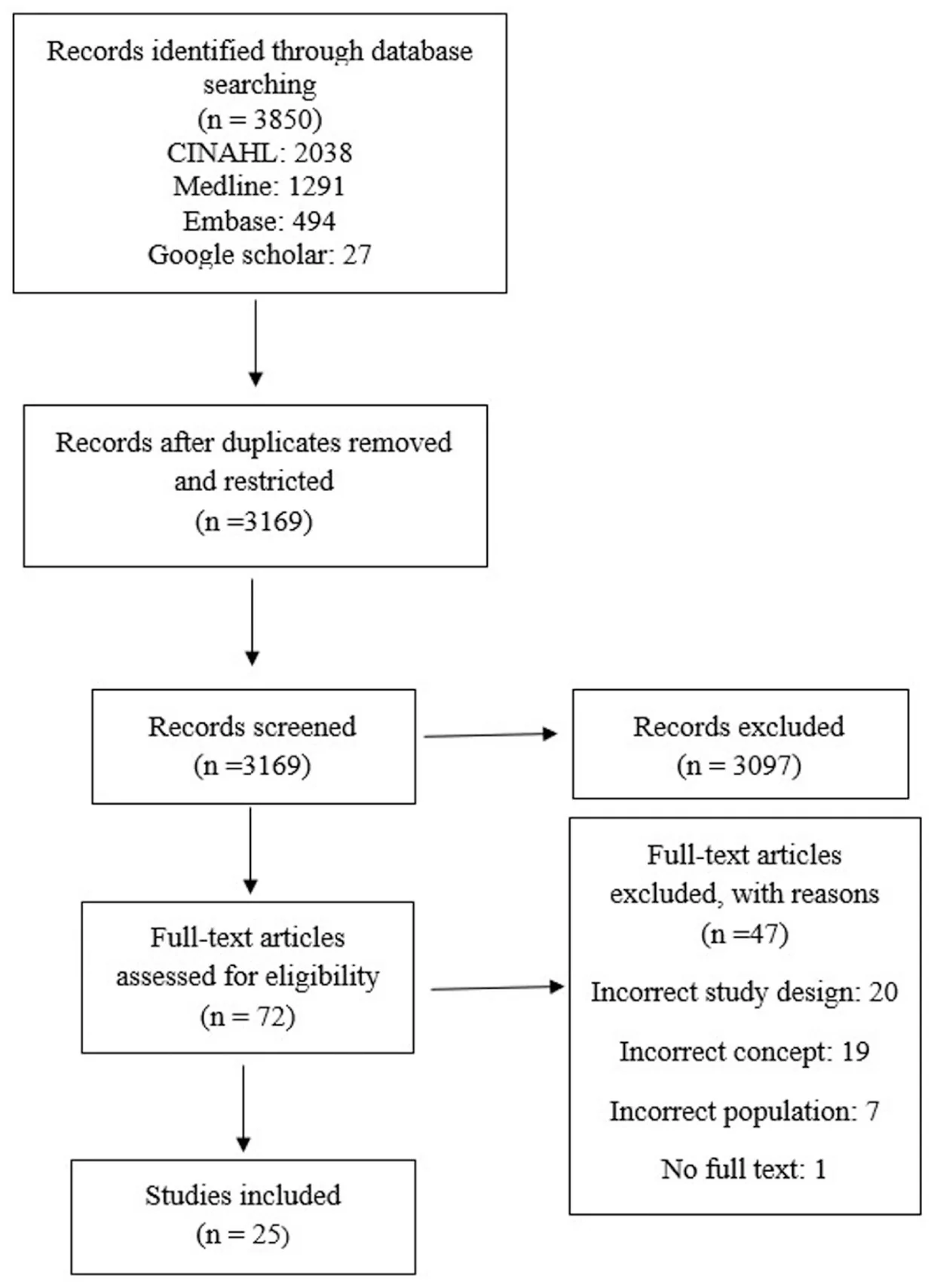
PRISMA Literature Search and Study Selection.

A total of 3850 abstracts were retrieved from the search strategy. After removing duplicates, 3169 records remained. The titles and abstracts of 3169 records were screened for relevance. Of these, 72 were determined to warrant full-text review. Following the application of the inclusion criteria, 47 papers were eliminated, leaving 25 studies for review. A record of the search procedure is presented in the PRISMA chart shown in Figure 1.

#### The Search Strategy and Process

The scoping review process was conducted between January 10, 2024, and January 31, 2024. A time limit was not imposed on this search to ensure all potential instruments are considered. A range of databases, including the Cumulative Index of Nursing & Allied Health Literature (CINAHL), MEDLINE, and Excerpta Medica database (Embase), were searched. These databases were chosen as they were relevant to the research aim, covering a broad area of medical, nursing, and midwifery literature. The Cochrane Database for Systematic Reviews was searched for previous reviews evaluating the psychometric properties of existing breastfeeding instruments by the COSMIN checklist, but none were found.

We constructed a search strategy with the support of a health sciences librarian. The following search terms and keywords were used: lactat* OR “breast feed*” OR “breast milk” OR “milk ejection” OR “ breast milk express” OR “ milk, human” OR “maternal milk” OR “infant nutrition” OR “infant feeding” combined using AND Boolean operator with “questionnair* and survey*” OR questionnair* OR instrument OR scale OR tool combined using AND Boolean operator with “properti* measure*” OR valid* OR reliabil* OR psychometric* OR reproducibil*. We also explored the reference lists of identified papers and the search engine Google Scholar for additional missing articles. The final search strategy for MEDLINE can be found in supplementary file 1.

#### Selection of Sources of Evidence

The final search results were exported into EndNote, and duplicates were removed. Then all citations were imported into Covidence for screening and eligibility determination. Two researchers (SM, a PhD-qualified midwife, and VV, a PhD-qualified psychologist) with expertise in scoping reviews examined the titles and abstracts in Covidence independently, and those that did not match the inclusion criteria were eliminated. Following the title and abstract screening, the full text was sourced for the remaining studies. Full-text articles were then assessed against the study objectives, again independently by the same two researchers. Any disagreements in any screening phase were resolved through discussion or the opinion of a third researcher (LS), a PhD-qualified midwife who is also an expert in scoping reviews.

### Evaluate the Measurement Properties

The measurement properties of the instruments included in the review were evaluated using the COSMIN methodology (Prinsen et al., 2018). The COSMIN methodology provides a structured and transparent framework for evaluating the quality of patient-reported outcome measures (Prinsen et al., 2018). Its purpose is to support informed selection of measurement instruments by examining whether an instrument is methodologically robust, whether its measurement properties are adequate, and the strength of the overall body of evidence (Prinsen et al., 2018). Using this approach, COSMIN was applied in three sequential steps, as described in the following sections (Prinsen et al., 2018):

#### Assessing Methodological Quality

To evaluate the methodological quality of each study related to a measurement property, we used the COSMIN risk of bias checklist (Mokkink et al., 2018). This checklist assesses the methodological quality of developing an instrument and its measurement properties, including structural validity, cross-cultural validity/measurement invariance, criterion validity, hypothesis testing, test-retest reliability, internal consistency, measurement error, and responsiveness. During this step, the studies were rated on a scale from “very good” to “inadequate” based on this checklist (Mokkink et al., 2018).

#### Rating Study Results

The measurement properties of each study were then evaluated based on the updated criteria for good measurement properties. The results were rated as “sufficient” (+), “insufficient” (-), or “inconsistent” (±) for content validity, and as “sufficient” (+), “insufficient” (-), or “indeterminate” (?) for other measurement properties (Prinsen et al., 2018).

#### Grading Quality of the Evidence

The quality of evidence was then graded using a modified GRADE approach (Prinsen et al., 2018). Initially, the evidence presented was assumed to be of high quality. However, the quality of this evidence was adjusted downwards by one or two levels based on certain factors such as the risk of bias, inconsistency, indirectness, and imprecision. These downgrades occurred if there was evidence of bias due to low study quality, unexplained inconsistencies in the study results, indirect results, or imprecision due to insufficient sample size (Guyatt et al., 2011; Prinsen et al., 2018). As a result, the evidence was categorised as moderate, low, or very low quality. In cases where the overall rating for a single measurement property was indeterminate (?), it would not be possible to assess the quality of the evidence (Prinsen et al., 2018). Each instrument was considered separately, and two reviewers (SM and KS) assessed the quality independently to ensure accuracy and reliability. Any disagreements were resolved through discussion and consensus.

### Select an Instrument

The COSMIN guidelines were used to categorise instruments into three groups (Prinsen et al., 2018). The groups are (A) instruments that have sufficient content validity and internal consistency for use, (B) instruments that require further validation studies, and (C) instruments that have high-quality evidence indicating insufficient measurement property (Prinsen et al., 2018). These categories help in selecting the most appropriate instruments for the population and construct of interest. Instruments classified as (A) can be recommended for broad use. Those categorised as (B) have the potential for recommendation but require further research to assess their quality. Instruments falling under category (C) should not be recommended for use (Xia et al., 2023).

## Results

### Characteristics of the Included Studies

The general characteristics of the included studies are shown in Table 1. Research activity in developing breastfeeding assessment instruments has increased, with the majority (63%) of studies conducted after 2010. The included studies covered various constructs, each focusing on different aspects of breastfeeding experiences, practices, and perceptions. Breastfeeding support and self-efficacy were the two constructs that were mentioned most frequently and remained consistent over time. New assessment instruments like the Breastfeeding Literacy Assessment Instrument (BLAI) (Valero-Chillerón et al., 2023) and the Breastfeeding Health Literacy Scale (BFHLS) (Chou et al., 2024) show that health literacy has recently become a key construct of interest.

**Table 1.** Characteristics of the Included Studies.

Author(s) (year) country	Name of instrument	Construct	Subscale	Target population	Number of items	Response options	Range of total score	Time to complete	Language	Psychometric proprieties reported
Abbass-Dick et al. (2020) Canada	Comprehensive Breastfeeding Knowledge Scale (CBKS)	Breastfeeding knowledge	(1) Managing milk supply (2) Persisting through challenges (3) Correcting misconceptions	Expectant parents	28	Three-option response set (disagree, unsure, agree) with scores reverse-coded for certain items	N/A	N/A	English	Concurrent validity, predictive validity, EFA, IC
Byrne and Arbon (2001) Australia	Breastfeeding Questionnaire	Breastfeeding support	(1) Support accessed, (2) healthcare practices, (3) maternal psychological perceptions (4) Breastfeeding history	Women with children between the ages of 1 to 3 years	32	N/A	N/A	N/A	English	Face validity, content validity, test-retest, Kappa, Pearson’s r correlation coefficient
Čatipović et al. (2023) Croatia	Parental Breastfeeding Behaviour and Attitude Questionnaire (BBAQ)	Breastfeeding behaviour and attitudes	(1) behaviour: (exclusive breastfeeding/supplemental feeding, breastfeeding duration, father’s support, method of breastfeeding), (2) attitude: (breastfeeding in public, partner support, misconceptions and denials, breastfeeding in the maternity ward, duration of breastfeeding)	Parents of children aged two to five years	32	The behaviour items are answered with ‘true’ or ‘false’. The attitude items use a Likert scale from 1 (strongly disagree) to 5 (strongly agree)	N/A	N/A	Croatian	Content validity, construct validity, discriminant validity, Known-group validity, IC
Charlesworth et al. (2023) Australia	Breastfeeding subjective norms scale	Subjective norms	(1) Breastfeeding in general (2) Breastfeeding in public	Couples expecting their first child	16	5-point Likert scale from 1 (strongly disagree) to 5 (strongly agree)	N/A	N/A	English	Construct validity, Concurrent validity, Predictive validity, IC
Chou et al. (2024) Taiwan	Breastfeeding Health Literacy Scale (BFHLS)	Breastfeeding health literacy	(1) functional (2) communicative (3) decision-making	Pregnant women	20	5-point Likert scale from 1 (strongly disagree) to 5 (strongly agree)	20 to 100	Approximately 10 minutes	Mandarin	Content validity, EFA, CFA, known-group validity, IC
Dennis and Faux (1999) Canada	Breastfeeding Self-Efficacy Scale (BSES)	Breastfeeding self-efficacy	(1) Technique (2) Intrapersonal (3) Thoughts	New breastfeeding mothers	32	5-point Likert scale from 1 (not at all confident) to 5 (always confident)	32 to 160	Less than 10 minutes	English	Content validity, construct validity, predictive validity, IC
Gökçe İsbir et al. (2024) Turkey	Breastfeeding Expectations Scale-A (BES-A)	Breastfeeding expectations	(1) Enthusiastic expectations of breastfeeding (2) Fearful/anxious expectations of breastfeeding	Pregnant women	12	5-point scale with various adjective pairs (relaxed–stressed; willing–unwilling; fresh–tired) indicating degrees of positive to negative expectations	Lower scores indicate higher positive expectations about breastfeeding	N/A	Turkish	Content validity, construct validity, EFA, CFA, IC
Hill and Humenick (1996) United States of America	H & H Lactation Scale	Satisfaction and confidence	(1) Maternal confidence/commitment to breastfeeding, (2) perceived infant breastfeeding satiety, (3) maternal-infant breastfeeding satisfaction	Breastfeeding mother	20	7-point Likert scale from 1 (strongly disagree) to 7 (strongly agree)	N/A	N/A	English	Content validity, concurrent validity, conceptual validity, predictive validity, IC
Hughes (1984) United States of America	Hughes Breastfeeding Support Scale (HBSS)	Breastfeeding support	(1) Emotional, (2) instrumental, (3) informational support	Breastfeeding primipara	30	Likert-type scale from 1 (none at all) to 4 (as much as I wanted)	N/A	Approximately 10 minutes, with a range from 5 to 20 minutes	English	Face validity, split half reliability, IC
Janke (1994) United States of America	Breast-feeding Attrition Prediction Tool (BAPT)	Attitude, subjective norms, perceived control	(1) Negative breastfeeding sentiment, (2) social and professional support, (3) breastfeeding control, (4) positive breastfeeding sentiment	Postpartum women who planned to breastfeed for at least 6 weeks	51	6-point summated rating scale	N/A	N/A	English	Content validity, EFA, known-group validity, predictive validity, discriminant validity, IC
Kelley et al. (1993) United States of America	Gender-Role Attitudes Toward Breast-Feeding	Gender-role attitudes	N/A	Breastfeeding primipara	6	N/A	N/A	N/A	English	CFA, known-groups validity, IC
Krishnamoorthy et al. (2022) India	Knowledge About Breastfeeding Benefits and Practices	Breastfeeding knowledge	(1) General benefits of breastfeeding, (2) breastfeeding practices, (3) specific hormonal benefits associated with breastfeeding	Antenatal and postnatal mothers	11	N/A	N/A	N/A	Tamil	Face validity, content validity, EFA, CFA, IC
Lakshman et al. (2011) United Kingdom	Maternal Attitudes Towards Infant Growth and Milk Feeding Practices	Attitudes	(1) Type of milk feeding, decision-making, and sources of advice, (2) frequency and quantity of feeds, (3) attitudes to infant feeding and growth, (4) theory-based beliefs about following infant feeding recommendations	Breastfeeding mothers	57	Multiple choice and 5-point Likert scale	N/A	N/A	English	Content validity, criterion validity, test-retest
Lau et al. (2017) Hong Kong	Breastfeeding Self-Regulation Questionnaire (BSRQ)	Maternal self-determination	(1) Autonomous motivation, (2) controlled motivation	Pregnant women	22	5-point Likert scale, from “completely disagree” to “completely agree”	N/A	N/A	English	Content validity, EFA, CFA, predictive validity, known-groups validity, test-retest, IC
Leff et al. (1994) United States of America	Maternal Breastfeeding Evaluation Scale (MBFES)	Breastfeeding experience	(1) Maternal enjoyment/role attainment, (2) infant satisfaction/growth, (3) lifestyle/maternal body image	Women who had breastfed at least one child	30	5-point Likert scale, from “strongly disagree” to “strongly agree”	N/A	10–15 minutes	English	Content validity, EFA, test-retest, IC
McKinley et al. (2019) United States of America	Prenatal Rating of Efficacy in Preparation to Breastfeed Scale (PREP to BF Scale)	Breastfeeding self-efficacy	(1) Individual processes, (2) interpersonal processes (3), professional advice, (4) social support	Pregnant women	39	10-point scale ranging from 0 (“Cannot do”) to 10 (“Highly certain can do”)	0 to 390	11–20 minutes	English	Face validity, content validity, EFA, CFA, test-retest, IC
Nanishi et al. (2021) Japan	Breastfeeding Support Scale	Breastfeeding support	(1) Support from breastfeeding peers and professional persons, (2) practical help, (3) emotional support and information from people the mother can rely on	Mothers up 3 months after birth	11	5-point Likert scale from 1 (“Not agree”) to 5 (“Agree”)	N/A	5 minutes	English	Content validity, face validity, factor analysis, known-groups validity, IC
Nommsen-Rivers and Dewey (2009) United States of America	Infant Feeding Intentions (IFI) Scale	Breastfeeding intentions	N/A	Pregnant mothers	5	Likert scale from 0 to 4 for	0 to 16	N/A	English	Content validity, construct validity, IC
Palmér and Jutengren (2019) Sweden	Existential Breastfeeding Difficulty Scale (ExBreastS)	Difficulty with initiating breastfeeding	(1) Mother-child interdependency, (2) exposure and vulnerability, (3) security and trust	Breastfeeding mothers	16	5-point Likert scale, from “strongly disagree” to “strongly agree”	N/A	N/A	Swedish	Face validity, content validity, EFA, IC
Stockdale et al. (2008) United States of America	Breastfeeding Motivational Instructional Measurement Scale (BMIMS)	Breastfeeding confidence	(1) Overall value of breastfeeding, (2) perceived midwife support, (3) expectancy to succeed	Women who had initiated breastfeeding	34		N/A	20 minutes	English	Face validity, content validity, EFA, IC
Valero-Chillerón et al. (2023) Spain	Breastfeeding Literacy Assessment Instrument (BLAI)	Breastfeeding literacy	(1) Access, (2) understand, (3) appraise, (4) apply breastfeeding-related information	Women during the perinatal period	26	A Likert-type scale with four response options	N/A	N/A	Spanish	Content validity, EFA, IC
Wells et al. (2006) United States of America	Prenatal Breastfeeding Self-Efficacy Scale	Breastfeeding self-efficacy	(1) Confidence regarding the skills and demands required for breastfeeding or extracting breastmilk using a breast pump, (2) confidence regarding gathering information about how to breastfeed, (3) confidence regarding breastfeeding around other people and feelings of embarrassment during breastfeeding, (4) confidence regarding social pressure when breastfeeding	Pregnant women	20	5-point Likert scale ranging from 1 to 5 (not at all sure, slightly sure, fairly sure, very sure, and completely sure)	20 to 100	N/A	English	Content validity, EFA, group differences, IC
Wood et al. (2022) United States of America	Breastfeeding Relationship Scale (BFRS)	Mother–infant mutual responsiveness	(1) Mother–infant breastfeeding interaction, (2) perceived adequate milk supply, (3) breastfeeding synchronicity	Mothers within 1–2 weeks postpartum	16	5-point Likert scale from “strongly disagree” to “strongly agree,” and a 5-point Likert scale, presumably from “never” to “always”	16 to 80	N/A	English	Content validity, CFA, IC
J.-L. Wu et al. (2023) China	The Mothers’ Breastfeeding Behaviour Scale within 6 Weeks Postpartum (MBBC-6W)	Breastfeeding behaviour	(1) Self-decision behaviour, (2) self-coping behaviour, (3) self-control behaviour, (4) resource coordination behaviour, (5) resource acquisition behaviour, (6) breastfeeding operation skills, (7) breastfeeding self-perception	Mothers within 6 weeks postpartum	36	5-point Likert scale (1 = it is strongly true about me, 2 = it is true about me, 3 = uncertain, 4 = it is false about me, 5 = it is strongly false about me)	36 to 180	N/A	Chimes	Content validity, EFA, convergent validity, distinguish validity, calibration validity, split-half reliability, test-retest, IC
Y. Wu et al. (2021) China	Breastfeeding Competency Scale (BCS)	Breastfeeding competency	(1) Breastfeeding knowledge, (2) Self-concept and psychology, (3) management of breast milk, (4) breastfeeding skill	Pregnant women	38	5-point Likert-type scale (from 1 = completely no corresponding to 5 = completely corresponding)	N/A	N/A	Chinese	Content validity, EFA, CFA, item analysis, split-half reliability, IC

EFA: Exploratory factor analysis; IC: internal consistency; CFA: confirmatory factor analysis; N/A: not applicable. Tools created in non-English languages were translated and published in English.

Most studies targeted pregnant and postpartum mothers, with a few studies focusing specifically on parents, such as the Comprehensive Breastfeeding Knowledge Scale (CBKS) (Abbass-Dick et al., 2020), Parental Breastfeeding Behaviour and Attitude Questionnaire (BBAQ) (Čatipović et al., 2023), and the Breastfeeding Subjective Norms Scale (Charlesworth et al., 2023). The range of items across the instruments varied from 5 on the Infant Feeding Intentions (IFI) Scale (Nommsen-Rivers & Dewey, 2009) to 57 items on the Maternal Attitudes Towards Infant Growth and Milk Feeding Practices Scale (Lakshman et al., 2011). Only seven of the instruments explicitly mentioned the time required to complete them, including the Breastfeeding Motivational Instructional Measurement Scale (BMIMS) (Stockdale et al., 2008), the Hughes Breastfeeding Support Scale (HBSS) (Hughes, 1984), Breastfeeding Self-Efficacy Scale (BSES) (Dennis & Faux, 1999), BFHLS (Chou et al., 2024), Maternal Breastfeeding Evaluation Scale (MBFES) (Leff et al., 1994), Prenatal Rating of Efficacy in Preparation to Breastfeed Scale (PREP to BF Scale) (McKinley et al., 2019), and the Breastfeeding Support Scale (Nanishi et al., 2021). Eight of the tools were developed in non-English languages and translated into English for publication. The remaining 17 were developed in English, and the United States is the most represented location for instrument development.

### Evaluate the Measurement Properties of Breastfeeding Instruments

We used the 3-step COSMIN approach as follows: Step 1 (assessment of methodological quality) was used for both instrument development and measurement properties, except for content validity, as the characteristics of instrument development were already incorporated into the overall evaluation of content validity (Mokkink et al., 2018). Steps 2 (evaluation of results) and 3 (grading the quality of evidence) were used for measurement properties only (Mokkink et al., 2018). This approach was followed throughout the results section for each relevant property and is summarised in Table 2. No instrument reported cross-cultural validity/measurement invariance, measurement error, or responsiveness.

**Table 2.** Quality of the Evidence for Measurement Properties of the Instruments.

**Instrument**	**Instrument Development**	**Validity**														**Reliability**									
		Content validity		Structural validity			Cross-cultural validity			Hypotheses testing			Criterion validity			Test-retest			Internal consistency			Measurement error			
		**R**	**Q**	**MQ**	**R**	**Q**	**MQ**	**R**	**Q**	**MQ**	**R**	**Q**	**MQ**	**R**	**Q**	**MQ**	**R**	**Q**	**MQ**	**R**	**Q**	**MQ**	**R**	**Q**	**Recommendation**
Comprehensive Breastfeeding Knowledge Scale (CBKS) (Abbass-Dick et al., 2020)	I	+	M	A	+	H		?	N	A	+	H		?	N		?	N	I	-	L		?	N	B
Breastfeeding Questionnaire (Byrne & Arbon, 2001)	I	**±**	**V**		?	N		?	N		**?**	N		?	N	D	**?**	M		**?**	N		?	N	B
Parental Breastfeeding Behaviour and Attitude Questionnaire (BBAQ) (Čatipović et al., 2023)	D	±	M	D	?	M		?	N	A	+	M		?	N		?	N	V	+	M		?	N	B
Breastfeeding Subjective Norms Scale (Charlesworth et al., 2023)	I	±	L	A	?	M		?	N		?	N	V	-	M		?	N	V	+	M		?	N	B
Breastfeeding Health Literacy Scale (BFHLS) (Chou et al., 2024)	D	+	H	V	+	H		?	N	A	+	H		?	N		?	N	V	+	H		?	N	A
Breastfeeding Self-Efficacy Scale (BSES) (Dennis & Faux, 1999)	I	+	H	D	-	L		?	N	A	+	H		?	N		?	N	V	+	H		?	N	A
Breastfeeding Expectations Scale-A (BES-A) (Gökçe İsbir Iet al., 2024)	I	+	H	V	+	H		?	N	A	+	H		?	N		?	N	V	+	H		?	N	A
H & H Lactation (Hill & Humenick, 1996)	I	±	V	I	-	L		?	N	A	+	H		?	N	D	?	L	V	+	H		?	N	B
Hughes Breastfeeding Support Scale (HBSS) (Hughes, 1984)	I	±	L		?	N		?	N		?	N		?	N		?	N	V	?	H		?	N	B
Breast-feeding Attrition Prediction Tool (BAPT) (Janke, 1994)	I	±	V	I	?	M		?	N	A	+	M		?	N		?	N	V	+	H		?	N	B
Gender-Role Attitudes Toward Breastfeeding (Kelley et al., 1993)	I	-	V	D	-	M		?	N	A	+	M		?	N		?	N	V	?	M		?	N	C
Knowledge About Breastfeeding Benefits and Practices (Krishnamoorthy et al., 2022)	I	±	L	V	?	H		?	N		?	N		?	N		?	N	V	?	H		?	N	B
Maternal Attitudes Towards Infant Growth and Milk Feeding Practices (Lakshman et al., 2011)	D	+	M		?	N		?	N		?	N		?	N	D	?	M	D	?	M		?	N	B
Breastfeeding Self-Regulation Questionnaire (BSRQ) (Lau et al., 2017)	I	±	M	V	+	H		?	N	A	+	M		?	N	I	?	M	V	+	H		?	N	B
Maternal Breastfeeding Evaluation Scale (MBFES) (Leff et al., 1994)	D	+	H	A	?	M		?	N		?	N		?	N	I	?	M	V	+	H		?	N	A
Prenatal Rating of Efficacy in Preparation to Breastfeed Scale (PREP to BF Scale) (McKinley et al., 2019)	I	±	L	I	?	L		?	N		?	N		?	N	I	?	M	V	+	H		?	N	A
Breastfeeding Support Scale (Nanishi et al., 2021)	I	±	M	D	?	M		?	N	A	+	H		?	N		?	N	V	+	H		?	N	B
Infant Feeding Intentions (IFI) Scale (Nommsen-Rivers & Dewey, 2009)	I	±	L		?	N		?	N		?	N		?	N		?	N	V	?	H		?	N	B
Existential Breastfeeding Difficulty Scale (ExBreastS) (Palmér & Jutengren, 2019)	D	±	L	V	?	H		?	N		?	N		?	N		?	N	V	+	H		?	N	B
Breastfeeding Motivational Instructional Measurement Scale (BMIMS) (Stockdale et al., 2008)	I	±	L	I	?	L		?	N		?	N		?	N		?	N	V	?	M		?	N	B
Breastfeeding Literacy Assessment Instrument (BLAI) (Valero-Chillerón et al. (2023)	I	±	M	V	?	H		?	N		?	N		?	N		?	N	V	+	H		?	N	B
Prenatal Breastfeeding Self-Efficacy (Wells et al., 2006)	I	+	L	V	?	M		?	N	A	+	M		?	N		?	N	I	+	M		?	N	B
Breastfeeding Relationship Scale (BFRS) (Wood et al., 2022)	I	±	V	V	+	H		?	N		?	N		?	N		?	N	V	+	H		?	N	B
The Mothers’ Breastfeeding Behaviour Scale within 6 Weeks Postpartum (MBBC-6W) (J.-L. Wu et al., 2023)	D	+	H	V	+	H		?	N		?	N		?	N		?	N	V	+	H		?	N	A
Breastfeeding Competency Scale (BCS) (Y. Wu et al., 2021)	*	±	M	V	+	H		?	N		?	N		?	N		?	N	V	+	H		?	N	B

MQ: methodological quality: V = very good; A = adequate; D = doubtful; I = inadequate information.

R: rating study results: + = sufficient; - = insufficient; ? = indeterminate; ± = inconsistent.

Q: Quality of evidence: H = high; M = moderate; L = low; VL = very low; N = not applicable.

* Empty cells indicate that a pilot test/cognitive interview (CI) study (or part of it) was not performed.

#### Instrument Development

The assessment of methodological quality of instrument development is structured around two aspects: instrument design and cognitive interviews or pilot tests. A few instruments, including the Existential Breastfeeding Difficulty Scale (ExBreastS) (Palmér & Jutengren, 2019), The Mothers’ Breastfeeding Behaviour Scale within 6 Weeks Postpartum (MBBC-6W) (J.-L. Wu et al., 2023), and the Breastfeeding Competency Scale (BCS) (Y. Wu et al., 2021), have achieved very good ratings in their design. Most instruments were rated as inadequate or doubtful due to a lack of samples representing the target population or the use of an inappropriate method for identifying relevant items (concept elicitation) during development.

Fourteen instruments conducted cognitive interviews or pilot tests with the target population during development. The results in these instruments were rated as doubtful or inadequate due to inappropriate methods used to assess comprehensibility and comprehensiveness of the scale instructions, items, response options, and recall period. When considering the total rating for instrument development, nearly all instruments were rated as inadequate. No instruments achieved a total score of very good or adequate.

#### Content Validity

Based on the evaluation of results, most instruments had inconsistent (±) rating scores due to unclear descriptions of relevance, comprehensiveness, and comprehensibility. The only instrument that received an insufficient (-) rating was the Gender-Role Attitudes instrument (Kelley et al., 1993).

Relevance was assessed by both mothers and professionals in the MBFES (Leff et al., 1994) and MBBC-6W (J.-L. Wu et al., 2023). Comprehensiveness was not evaluated with mothers in any study and was only assessed by professionals in the BBAQ (Čatipović et al., 2023) and Breastfeeding Support Scale (Nanishi et al., 2021). Comprehensibility was measured through feedback from either mothers or professionals in the BBAQ (Čatipović et al., 2023), BFHLS (Chou et al., 2024), BSES (Dennis & Faux, 1999), Breastfeeding Expectations Scale-A (BES-A) (Gökçe İsbir et al., 2024), HBSS (Hughes, 1984), MBFES (Leff et al., 1994), PREP to BF (McKinley et al., 2019), Breastfeeding Support Scale (Nanishi et al., 2021), IFI (Nommsen-Rivers & Dewey, 2009), ExBreastS (Palmér & Jutengren, 2019), and the BMIMS (Stockdale et al., 2008).

Based on grading the quality of evidence, almost one-third of the instruments had low-quality evidence for content validity. In contrast, five instruments exhibited high-quality evidence, including the BFHLS (Chou et al., 2024), BSES (Dennis & Faux, 1999), BES-A (Gökçe İsbir et al., 2024), MBFES (Leff et al., 1994), and MBBC-6W (J.-L. Wu et al., 2023).

#### Other Types of Validity: Structural, Cross-Cultural, Hypothesis Testing, and Criterion Validity

All of the instruments demonstrated evidence of structural validity except for the Breastfeeding Questionnaire (Byrne & Arbon, 2001), HBSS (Hughes, 1984), and the IFI (Nommsen-Rivers & Dewey, 2009). Based on the methodological quality, the BFHLS (Chou et al., 2024), BES-A (Gökçe İsbir et al., 2024), Knowledge About Breastfeeding Benefits and Practices (Krishnamoorthy et al., 2022), Breastfeeding Self-Regulation Questionnaire (BSRQ) (Lau et al., 2017), ExBreastS (Palmér & Jutengren, 2019), BLAI (Valero-Chillerón et al., 2023), Prenatal Breastfeeding Self-Efficacy (Wells et al., 2006), Breastfeeding Relationship Scale (BFRS) (Wood et al., 2022), MBBC-6W (J.-L. Wu et al., 2023), and BCS (Y. Wu et al., 2021) received a very good COSMIN rating score due to their appropriate sample size and the use of both confirmatory and exploratory factor analysis. Among these instruments, the BFHLS (Chou et al., 2024), BES-A (Gökçe İsbir et al., 2024), BSRQ (Lau et al., 2017), BFRS (Wood et al., 2022), MBBC-6W (J.-L. Wu et al., 2023), and the BCS (Y. Wu et al., 2021) were rated sufficient (+) for the evaluation of results, because they met the required fit measures for factor analysis with reported comparative fit index (CFI) values >0.95 or root mean square error of approximation (RMSEA) <0.06 (Prinsen et al., 2018). Based on grading the quality of evidence, only four instruments had low-quality evidence for structural validity, including the BSES (Dennis & Faux, 1999), H & H Lactation (Hill & Humenick, 1996), PREP to BF Scale (McKinley et al., 2019), and BMIMS (Stockdale et al., 2008).

Eleven instruments assessed hypothesis testing for construct validity by comparing them with other outcome measurement instruments (convergent validity) or comparing them between subgroups (discriminative or known-groups validity) with adequate methodological quality. All these instruments received sufficient (+) scores for the evaluation of results, because their results aligned with the expected hypotheses. Based on grading the quality of evidence, none of the instruments had low-quality evidence for construct validity.

None of the instruments conducted cross-cultural validity/measurement invariance. Only one instrument, the Breastfeeding Subjective Norms Scale (Charlesworth et al., 2023), exhibited criterion validity, but its criterion validity score was insufficient (-) due to a correlation with the gold standard, which was less than 0.70 (Prinsen et al., 2018).

#### Reliability

Test-retest reliability was assessed in six instruments, with inadequate or doubtful methodological quality due to a lack of justification for the interval time, evidence of sample stability, or adequate reliability testing. Indeterminate rating score (?) for the evaluation of results was found in all instruments due to not reporting intraclass correlation (ICC) or weighted Kappa. Based on grading the quality of evidence, none of the instruments had high-quality evidence for test-retest reliability.

All instruments, except for the Breastfeeding Questionnaire (Byrne & Arbon, 2001), demonstrated evidence of internal consistency. Most instruments had very good methodological quality scores due to calculating internal consistency for each unidimensional scale or subscale separately and adequate statistical methods. A sufficient rating score (+) for the evaluation of results was found in most instruments due to Cronbach’s alpha(s) being ≥0.70 for each unidimensional scale or subscale (Prinsen et al., 2018). Based on grading the quality of evidence, only one instrument, the CBKS (Abbass-Dick et al., 2020), had low-quality evidence for internal consistency.

### Recommended Breastfeeding Instruments

Based on the evaluation results, six instruments, including the BFHLS (Chou et al., 2024), BSES (Dennis & Faux, 1999), BES-A (Gökçe İsbir et al., 2024), MBFES (Leff et al., 1994), Prenatal Breastfeeding Self-Efficacy (Wells et al., 2006), and MBBC-6W (J.-L. Wu et al., 2023), were rated as sufficient (+) for content validity, and their internal consistency was rated as sufficient (+). Thus, they were recommended for use under COSMIN category A. The Gender-Role Attitudes (Kelley et al., 1993) was rated insufficient (-) for content validity, so it was not recommended for use under COSMIN category C. The CBKS (Abbass-Dick et al., 2020) and Maternal Attitudes Towards Infant Growth and Milk Feeding Practices Scale (Lakshman et al., 2011) were sufficient (+) for content validity, but their internal consistency was rated as insufficient (-) or indeterminate (?). Thus, they were classified under COSMIN category B. The other instruments had evidence of “indeterminate (±)” content validity, so they were also classified under category B, indicating that further validation is required before they can be recommended for use.

## Discussion

In recent years, research has been focused on developing breastfeeding assessment instruments, particularly in high-income settings. Concerns about inadequate protection, promotion, and support for breastfeeding in these regions (Vaz et al., 2021) underscore the importance of accurate assessment tools in identifying gaps, monitoring breastfeeding practices, and creating evidence-based interventions tailored to these specific challenges (Gardona & Barbosa, 2018).

In this review, two instruments, BLAI (Valero-Chillerón et al., 2023) and BFHLS (Chou et al., 2024), assessed breastfeeding health literacy. Health literacy, a relatively new construct in breastfeeding measurement, offers a wider perspective than traditional knowledge alone. Health literacy is defined as “an individual’s ability to obtain and translate knowledge and information to maintain and improve health in ways appropriate to the individual and community context” (Liu et al., 2020, p. 7). While health literacy can be a protective factor for breastfeeding (Valero-Chillerón et al., 2022), there are limited studies examining mothers’ breastfeeding behaviours and their health literacy (Toksoy & Cesur, 2020). Developing breastfeeding health literacy tools can help bridge this gap and provide valuable insights into the connection between health literacy and breastfeeding practices.

It is crucial for the development of an instrument to include relevant items that align with the concepts being measured (Husbands et al., 2020). Empirical work to identify relevant items will be referred to as “concept elicitation” (Husbands et al., 2020). According to the COSMIN guideline, qualitative empirical research with the target population is necessary for effective concept elicitation (Terwee et al., 2018). This review underscores the importance of this principle, as it reveals that most breastfeeding instruments did not use qualitative methods to identify their items. Items in breastfeeding instruments need to be relevant and comprehensive and easy to understand for the study population (Xia et al., 2023). Pilot tests with the target population are vital to confirm these elements (Mokkink et al., 2010). However, only 14 studies of breastfeeding instruments included a pilot test with the target population, and all of these lacked detail.

Asking patients and professionals about the comprehensiveness of the items, response options, and instructions is part of evaluating content validity (Terwee et al., 2018). However, only two breastfeeding instruments sought expert advice regarding comprehensiveness, and none consulted the target population. The quality of an instrument heavily depends on adequate content validity assessment (Terwee et al., 2018), so instruments with enough content validity can be recommended for broad use (Mokkink et al., 2018). This review showed that most breastfeeding instruments need more content validity assessment. Only six had sufficient content validity and were recommended for use under category A. All these instruments, except MBFES (Leff et al., 1994), were published after 2018, possibly due to the influence of COSMIN guidelines and checklists introduced in the same year. Additionally, the availability of new psychometric techniques in recent years has contributed to the enhanced development processes of these newer instruments. Breastfeeding self-efficacy is a key predictor of breastfeeding duration and exclusivity (Titaley et al., 2021). Two of the six instruments in category A are related to breastfeeding self-efficacy, including the Breastfeeding Self-Efficacy Scale (BSES) and Prenatal Breastfeeding Self-Efficacy. While this review focused on the original versions of breastfeeding questionnaires, it is important to note that the Short Form of the Breastfeeding Self-Efficacy Scale (BSES-SF) is the most feasible tool due to its brevity and ease of use (Borona et al., 2023).

After evaluating content validity, the next step is to evaluate the internal structure of an instrument (Terwee et al., 2018). Internal structure describes the relationships between various items in an instrument and how they can be grouped into a scale or subscale (Mokkink et al., 2018). This step involves evaluating structural validity through factor analyses, item response theory (IRT) or Rasch analyses, cross-cultural validity/measurement invariance, and internal consistency (Mokkink et al., 2018). Confirmatory factor analysis (CFA) and exploratory factor analysis (EFA) were performed on six breastfeeding instruments, including the BFHLS (Chou et al., 2024), BES-A (Gökçe İsbir et al., 2024), BSRQ (Lau et al., 2017), BFRS (Wood et al., 2022), MBBC-6W (J.-L. Wu et al., 2023), and the BCS (Y. Wu et al., 2021). They met the required fit measures for factor analysis, which effectively represent the theoretical concepts of breastfeeding (Prinsen et al., 2018). None of the breastfeeding instruments conducted cross-cultural validity/measurement invariance, which is essential to ensure the accuracy of the instrument among culturally diverse populations (Lee et al., 2020). The COSMIN interprets “culturally diverse populations” broadly, encompassing not only ethnicity or language groups but also groups differing by gender, age, or patient characteristics (Prinsen et al., 2018). Cross-cultural validity evaluates whether a scale maintains consistency across these groups by assessing measurement invariance (MI) (Prinsen et al., 2018). The multi-group confirmatory factor analyses (MGCFA) are often used to assess measurement invariance and ensure comparability across diverse groups (Milfont & Fischer, 2010; Prinsen et al., 2018). However, in the original scales included in the review, there was no explicit assessment of measurement invariance. Although cross-cultural validity may have been assessed in later translation or adaptation studies, this review only included the original versions of the breastfeeding scales. Therefore, further studies are necessary to confirm the validity of breastfeeding instruments across different cultures and languages. Internal consistency should be conducted to evaluate Cronbach’s alpha for each dimension of breastfeeding instruments (Lee et al., 2020; Mokkink et al., 2010), whereas CBKS (Abbass-Dick et al., 2020) reported Cronbach’s alpha for the total instrument. Moreover, the sample sizes of studies assessing the internal consistency of CBKS (Abbass-Dick et al., 2020) and Maternal Attitudes Towards Infant Growth and Milk Feeding Practices Scale (Lakshman et al., 2011) were below 100, so further studies are required to evaluate the internal consistency of these two breastfeeding instruments. Sufficient internal consistency is crucial for recommending the use of an instrument. These two instruments, because of insufficient internal consistency, are categorised in group B, so they require further research to assess their quality (Mokkink et al., 2018).

It is essential to ensure the reliability, minimal measurement error, and sensitivity to changes in breastfeeding instruments for their widespread use (Xia et al., 2023). However, only six breastfeeding instruments were evaluated for test-retest reliability, and their intraclass correlation values were not documented, indicating unsatisfactory reliability. None of the breastfeeding instruments were assessed for measurement error, including systematic and random errors in ratings that are not due to actual changes in what is being measured (Mokkink et al., 2018). Furthermore, none of the instruments have been examined for responsiveness, likely due to the absence of longitudinal validation, including intervention studies (Cheng et al., 2022). Therefore, it is crucial to conduct further research to assess the responsiveness and measurement error of these instruments in the future.

### Strengths and Limitations

A scholarly librarian supported the publication search to ensure the accuracy of search terms. Two reviewers independently reviewed all papers for inclusion, with the support of a third reviewer to address conflicts. This ensured that all relevant papers were included. Using COSMIN facilitated a comprehensive evaluation of breastfeeding instruments and provided valuable insights about the quality of available measures. Two reviewers also independently assessed the quality to ensure accuracy and reliability.

The scoping review has some limitations that need to be acknowledged. Limitations include potential oversight of relevant non-English studies due to language constraints and the reliance on specific databases, which might have led to missing tools developed and validated in other languages. Another limitation of this review is that we only included the original versions of the scales to assess their psychometric properties. This may have excluded useful information from subsequent adaptations and refinements of these scales in later studies. In this study, interpretability and feasibility were not evaluated, so future research is suggested to assess these properties.

## Conclusion

This scoping review summarised the psychometric properties of the available breastfeeding instruments. Based on the COSMIN guideline, six breastfeeding instruments were considered reliable for use, while the Gender-Role Attitudes instrument (Kelley et al., 1993) is not recommended. The study also highlights the need for further research into the measurement properties of existing breastfeeding instruments, especially cross-cultural validity/measurement invariance, measurement error, and criteria validity. Additionally, researchers are encouraged to develop novel breastfeeding instruments guided by the COSMIN framework.

## Supplemental Material

sj-pdf-1-jhl-10.1177_08903344261430247 – Supplemental material for Measurement Properties and Quality of Maternal Breastfeeding Instruments: A Scoping Review Supplemental material, sj-pdf-1-jhl-10.1177_08903344261430247 for Measurement Properties and Quality of Maternal Breastfeeding Instruments: A Scoping Review by Seyedeh Samira Mokhlesi, Vidanka Vasilevski, Kolsoom Safari and Linda Sweet in Journal of Human Lactation
