# Supplementary material for: Measurement Properties and Quality of Maternal Breastfeeding Instruments: A Scoping Review
Source: J Hum Lact. 2026 Apr 26;42(3):781–94. doi: 10.1177/08903344261430247 (PMC13615321; doi:10.1177/08903344261430247)
Supplement: Supplementary file 1 [file sj-pdf-1-jhl-10.1177_08903344261430247.pdf]

EBSCOhost

Wednesday, January 31, 2024 3:42:37 AM

#	Query	Limiters/Expanders	Last Run Via	Results
S28	S15 AND S26 AND S27	Search modes - Boolean/Phrase	Interface - EBSCOhost Research Databases<br>Search Screen - Advanced Search<br>Database - MEDLINE Complete	1,291
S27	S21 OR S22 OR S23 OR S24 OR S25	Search modes - Boolean/Phrase	Interface - EBSCOhost Research Databases<br>Search Screen - Advanced Search<br>Database - MEDLINE Complete	Display
S26	S16 OR S17 OR S18 OR S19 OR S20	Search modes - Boolean/Phrase	Interface - EBSCOhost Research Databases<br>Search Screen - Advanced Search<br>Database - MEDLINE Complete	Display
S25	AB Reliabil* OR TI Reliabil*	Search modes - Boolean/Phrase	Interface - EBSCOhost Research Databases<br>Search Screen - Advanced Search<br>Database - MEDLINE Complete	Display
S24	AB Valid* OR TI Valid*	Search modes - Boolean/Phrase	Interface - EBSCOhost Research Databases<br>Search Screen - Advanced Search<br>Database - MEDLINE Complete	Display
S23	AB “Properti* measure*” OR TI “Properti* measure*”	Search modes - Boolean/Phrase	Interface - EBSCOhost Research Databases<br>Search Screen - Advanced Search<br>Database - MEDLINE Complete	Display
S22	AB Psychometric* OR TI Psychometric*	Search modes - Boolean/Phrase	Interface - EBSCOhost Research Databases<br>Search Screen - Advanced	Display

			Search<br>Database - MEDLINE Complete	
S21	(MM "Psychometrics")	Search modes - Boolean/Phrase	Interface - EBSCOhost Research Databases<br>Search Screen - Advanced Search<br>Database - MEDLINE Complete	Display
S20	AB questionnaire* OR TI questionnaire*	Search modes - Boolean/Phrase	Interface - EBSCOhost Research Databases<br>Search Screen - Advanced Search<br>Database - MEDLINE Complete	Display
S19	AB Scale OR TI Scale	Search modes - Boolean/Phrase	Interface - EBSCOhost Research Databases<br>Search Screen - Advanced Search<br>Database - MEDLINE Complete	Display
S18	AB Instrument OR TI Instrument	Search modes - Boolean/Phrase	Interface - EBSCOhost Research Databases<br>Search Screen - Advanced Search<br>Database - MEDLINE Complete	Display
S17	AB “Questionnair* and Survey*” OR TI “Questionnair* and Survey*”	Search modes - Boolean/Phrase	Interface - EBSCOhost Research Databases<br>Search Screen - Advanced Search<br>Database - MEDLINE Complete	Display
S16	(MM "Surveys and Questionnaires")	Search modes - Boolean/Phrase	Interface - EBSCOhost Research Databases<br>Search Screen - Advanced Search<br>Database - MEDLINE Complete	Display
S15	S1 OR S2 OR S3 OR S4 OR S5 OR S6 OR S7 OR S8 OR S9 OR S10 OR S11 OR S12 OR S13 OR S14	Search modes - Boolean/Phrase	Interface - EBSCOhost Research Databases<br>Search Screen - Advanced Search<br>Database - MEDLINE Complete	Display

S14	AB “Breast Milk express*” OR TI “Breast Milk express*”	Search modes - Boolean/Phrase	Interface - EBSCOhost Research Databases<br>Search Screen - Advanced Search<br>Database - MEDLINE Complete	Display
S13	AB Lactat* OR TI Lactat*	Search modes - Boolean/Phrase	Interface - EBSCOhost Research Databases<br>Search Screen - Advanced Search<br>Database - MEDLINE Complete	Display
S12	AB Breastmilk OR TI Breastmilk	Search modes - Boolean/Phrase	Interface - EBSCOhost Research Databases<br>Search Screen - Advanced Search<br>Database - MEDLINE Complete	Display
S11	AB Breatfed* OR TI Breatfed*	Search modes - Boolean/Phrase	Interface - EBSCOhost Research Databases<br>Search Screen - Advanced Search<br>Database - MEDLINE Complete	Display
S10	AB “Breast fed*” OR TI “Breast fed*”	Search modes - Boolean/Phrase	Interface - EBSCOhost Research Databases<br>Search Screen - Advanced Search<br>Database - MEDLINE Complete	Display
S9	AB “breast feed*” OR TI “breast feed*”	Search modes - Boolean/Phrase	Interface - EBSCOhost Research Databases<br>Search Screen - Advanced Search<br>Database - MEDLINE Complete	Display
S8	AB “Breastfeed*” OR TI “Breastfeed*”	Search modes - Boolean/Phrase	Interface - EBSCOhost Research Databases<br>Search Screen - Advanced Search<br>Database - MEDLINE Complete	Display
S7	AB “Breast milk” OR TI “Breast milk”	Search modes - Boolean/Phrase	Interface - EBSCOhost Research Databases<br>Search Screen - Advanced Search<br>Database - MEDLINE Complete	Display

S6	AB “Infant feed*” OR TI “Infant feed*”	Search modes - Boolean/Phrase	Interface - EBSCOhost Research Databases<br>Search Screen - Advanced Search<br>Database - MEDLINE Complete	Display
S5	AB “Mother* milk” OR TI “Mother* milk”	Search modes - Boolean/Phrase	Interface - EBSCOhost Research Databases<br>Search Screen - Advanced Search<br>Database - MEDLINE Complete	Display
S4	(MH "Breast Milk Expression")	Search modes - Boolean/Phrase	Interface - EBSCOhost Research Databases<br>Search Screen - Advanced Search<br>Database - MEDLINE Complete	Display
S3	(MM "Milk, Human")	Search modes - Boolean/Phrase	Interface - EBSCOhost Research Databases<br>Search Screen - Advanced Search<br>Database - MEDLINE Complete	Display
S2	(MH "Breast Feeding+")	Search modes - Boolean/Phrase	Interface - EBSCOhost Research Databases<br>Search Screen - Advanced Search<br>Database - MEDLINE Complete	Display
S1	(MH "Lactation+")	Search modes - Boolean/Phrase	Interface - EBSCOhost Research Databases<br>Search Screen - Advanced Search<br>Database - MEDLINE Complete	Display
